# The future of HRT/MHT and Alzheimer’s disease risk, onset and progression

**DOI:** 10.1016/j.tjpad.2026.100508

**Published:** 2026-02-13

**Authors:** Edwin D. Lephart, Dawson W. Hedges, Frederick Naftolin, Zoe D. Draelos

**Affiliations:** a Department of Cell Biology, Physiology and The Neuroscience Center, Brigham Young University, Provo, UT 84602, USA; b Department of Psychology (Cognitive Behavior) and The Neuroscience Center, Brigham Young University, Provo, UT 84602, USA; c Chief Scientific Officer, e-Bio-Corp, Woodbridge, CT 06525, USA; d emeritus Department of Obstetrics and Gynecology, NYU Langone Medical Center, NY, NY 10022, USA; e President, Dermatology Consulting Services, PLLC, High Point, NC 27261, USA

Before the Women’s Health Initiative (WHI; 2002) hormone replacement therapy (HRT) was widely prescribed, strongly endorsed, and broadly considered not just for menopausal symptom relief, but also as prevention to reduce the risk of: cardiovascular disease, osteoporosis, cognitive decline and helped maintain overall health. Prescribing patterns included women without significant menopausal symptoms, and many women stayed on HRT for years or decades. In brief, estrogen alone (for women without a uterus) and estrogen-progestin combinations were both widely used (Fig. 1) [1].

**Fig. 1:**
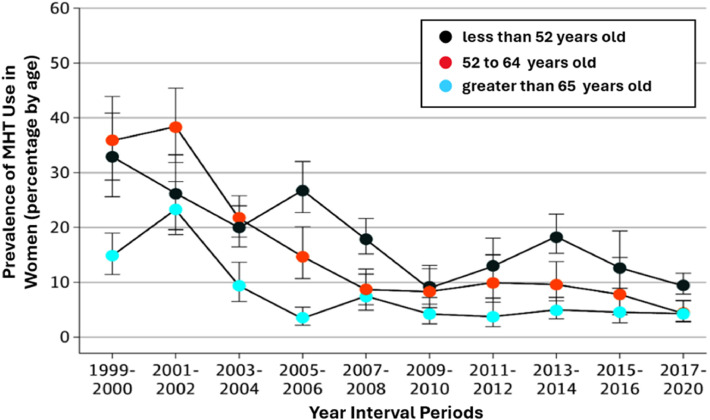
Patterns of Menopause Hormone Therapy (MHT) Use in Women in the United States by Age. Prevalence of MHT use was extracted from the prescription medication data collected during NHANES household interviews. Each data point (by age) and corresponding 95 % confidence interval are displayed. Adapted from Yang and Toriola, JAMA Health Forum, 2024, 5: e243128 by CC-BY license. Estrogen only formulations accounted for greater than fifty percent of HMT. Values before the Women’s Health Initiative (WHI) were approximately 40 % in 1992 (not shown), in 1999 26.9 % were recorded and post-WHI in 2020 approximately 4.7 % were calculated. In 1990s via the national prescription data showed approximately 90 million/year were recorded. While conjugated equine estrogen (CEE) + medroxyprogesterone acetate (MPA) were the predominate hormones utilized in the early years of Hormone Replacement Therapy (HRT), these have been largely superseded by new approved regulated formulations of estradiol along with various later generation progestogens and progesterone preparations.

The non-stratified for age WHI findings in 2002 showed that combined oral conjugated equine estrogen (CEE) - medroxyprogesterone therapy started on average ten years postmenopause was associated with increased risk of: breast cancer, stroke, blood clots, coronary heart disease, and CEE only therapy showed increased stroke and clot risk, but no cardiovascular benefit [1,2]. After the WHI (post 2002) report, the immediate impact was a massive drop in HRT use worldwide. Many women abruptly stopped therapy, while media coverage emphasized risks often without recognizing the inappropriate age and degree of disease in the initial WHI report [1,2]. HRT was no longer recommended for disease prevention but was framed as symptom-focused therapy primarily for vasomotor symptoms such as (hot flashes, night sweats) and genitourinary syndrome of menopause with guidance of use for the lowest effective dose for the shortest duration needed, best suited for symptomatic women generally under age 60 or within 10 years of menopause [1,2].

Later analyses showed that HRT risks were age- and time-dependent where recently menopausal women have a more favorable risk profile [2]. Furthermore, transdermal estrogen and lower-dose formulations may reduce some risks. In summary, before the WHI, HRT was broadly prescribed as a long-term health-preserving therapy, whereas, after WHI, HRT became cautiously and selectively used and focused on symptom relief than prevention [1,2]. However, studies before and since the WHI on appropriate aged women, continues to be prescribed in recently menopausal women.

Direct epidemiology prevalence of Alzheimer’s disease estimates specifically stratified by menopausal status are lacking (before vs. after 2002). However, overall trends showed a decrease in the U.S. between 1993 and 2002, from 12.2 to 8.8 %, whereas, by 2020 prevalence was 11 to 12 % with women making up about two-thirds of those cases [1].

Today, HRT [currently referred to as Menopause Hormone Therapy (MHT] has transitioned where The American College of Obstetricians and Gynecologists recommends low-dose topical estrogen for vaginal dryness/atrophy to dermatological use, where in 2023 at the Menopause Society meeting the use of local estrogen cream on the face as an effective treatment for aging skin was presented, leading several cosmetic/dermatology companies to furnish estrogen formulations as extemporaneously compounded products for aesthetic treatments [2]. Recently, HRT/MHT has been presented as a consideration for perimenopausal and menopausal patients for aesthetic treatment [3] and earlier studies supported by more recent reports showed the positive influences of HRT/MHT on collagen, skin thickness, elasticity, hydration and wound healing [2,3]. Notably, this has a new perspective since the US FDA (November 10, 2025) removed their stringent warning on hormone therapy products for menopausal women (reversing a 2003 decision) and stated the treatments potentially offer heart, brain and bone health benefits [4].

This transformation of scientific thought/practice has many implications in reference to neurodegenerative diseases, where the prevalence of Alzheimer’s disease (AD) is well documented with postmenopausal women accounting for over sixty percent of all those affected, which is only partially explained by survival rates and longevity parameters [2]. In this regard, HRT/MHT may potentially reduce the risk, onset and progression of AD, since a generation of women have been deprived of HRT/MHT that are known to reduce aging, neuroinflammation by maintaining the blood-brain-barrier, enhance DNA repair, mitochondrial, immune, and sleep cycle functions, decrease oxidative stress in astrocytes, microglia and neurons and diminish the incidence of hot flashes, amyloid beta plaques and tau protein phosphorylation that contribute to neuro-fibrillary tangles [2]. Together these factors define the pleiotropic actions of estrogen, which are essential to women’s health. Remarkably, in a recent review a positron emission tomography (PET) scan study was cited, which showed that postmenopausal women displayed the highest estrogen receptor expression in the brain when estrogen levels are lowest, suggesting that neuronal cells were searching for this steroid chemical signal a finding that may provide additional support for HRT/MHT to enhance brain health in women and potentially protect against AD [5].

The range of associations with HRT/MHT suggest that the potential use of this therapy in the future could have a significant impact on AD as women age during perimenopause and menopause [2,5]. Because menopause requires a multidisciplinary scientific and medical approach for optimal treatment, the wide range of HRT/MHT effects provides opportunities for different medical disciplines to collaborate and determine the benefits of HRT/MHT not only for neurodegenerative diseases like AD but also for further studies on parameters like cognitive function and depression [2]. This opens new horizons for gynecologists, neuroscientists, dermatologists and other physicians and scientists to collaborate in professional interchanges and investigational studies to advance women’s health, in general, to lead the way to develop the knowledge, timing and treatment of AD in women during aging [2,5].

Declaration of the use of generative AI and AI-assisted technologies in scientific writing and in figures, images and artwork- none were used.

## Funding

none

## CRediT authorship contribution statement

**Edwin D. Lephart:** Writing – review & editing, Writing – original draft, Supervision, Resources, Funding acquisition, Formal analysis, Conceptualization. **Dawson W. Hedges:** Writing – review & editing, Writing – original draft, Formal analysis. **Frederick Naftolin:** Writing – review & editing, Writing – original draft, Formal analysis. **Zoe D. Draelos:** Writing – review & editing, Formal analysis.

## Declaration of competing interest

The authors have no conflict of interest as stated in the Letter to the Editor
